# P-363. Adherence to Guideline Recommended HIV Care in Patients with an Elevated HIV Viral Load is Associated with Improved Rates of Virologic Suppression at One Year

**DOI:** 10.1093/ofid/ofaf695.581

**Published:** 2026-01-11

**Authors:** Kelli A Bagwell, John R Bassler, Emma Kay, Melanie C Goebel, laura Bamford, Lindsay Browne, Katerina Christopoulos, Barbara Gripshover, Kenneth H Mayer, Richard Moore, Michael J Mugavero, Thomas P Giordano

**Affiliations:** Baylor College of Medicine, Houston, Texas; University of Alabama at Birmingham, Birmingham, AL; University of Alabama at Birmingham, Birmingham, AL; Baylor College of Medicine, Houston, Texas; UCSD, san diego, California; UNC Chapel Hill, Chapel Hill, North Carolina; University of California San Francisco, San Francisco, CA; Case Western Reserve University, Cleveland, Ohio; Fenway Health/ Harvard Medical School, Boston, Massachusetts; Johns Hopkins University, Baltimore, MD; UAB, Birmingham, Alabama; Baylor College of Medicine, Houston, Texas

## Abstract

**Background:**

Guidelines recommend checking an HIV viral load (VL) within 4-8 weeks (wk) after starting or restarting anti-retroviral therapy (ART) and every 4-8 wk thereafter until virologic suppression (VS). HIV provider visits are recommended every 3-4 months until VS is reliably demonstrated.  We postulate that these early lab and provider visits are proxies of early retention in care (RIC). We sought to determine if adherence to these guidelines is associated with VS at one year in patients with an elevated VL.

**Figure:**
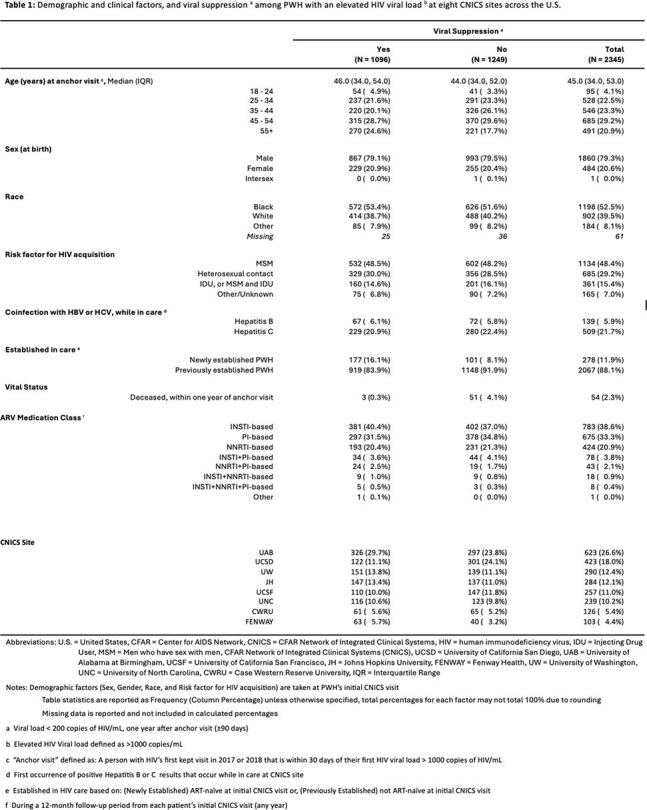

**Figure:**
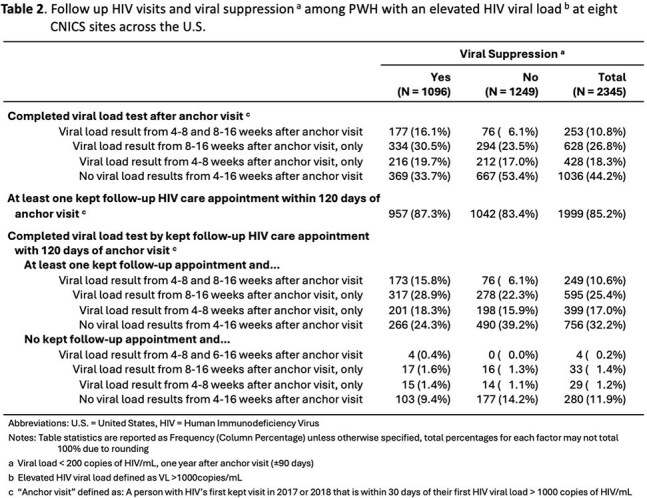

**Methods:**

We conducted a retrospective study of adult people with HIV (PWH) in 8 clinics in the Center for AIDS Research (CFAR) Network of Integrated Clinical Systems (CNICS) network with an HIV VL >1000c/mL in 2017 or 2018 (to avoid COVID-era changes), and a completed provider visit +/-30 days (d) of that VL, which became the anchor visit (AV) for the study. The primary outcome was VS (< 200c/mL) one year after the AV (+/-90 d). Explanatory variables in the multivariable model included having an HIV VL measured 4-8 wk after the AV, 8-16 wk after the AV and at least 1 provider visit within 120 d after the AV.

**Figure:**
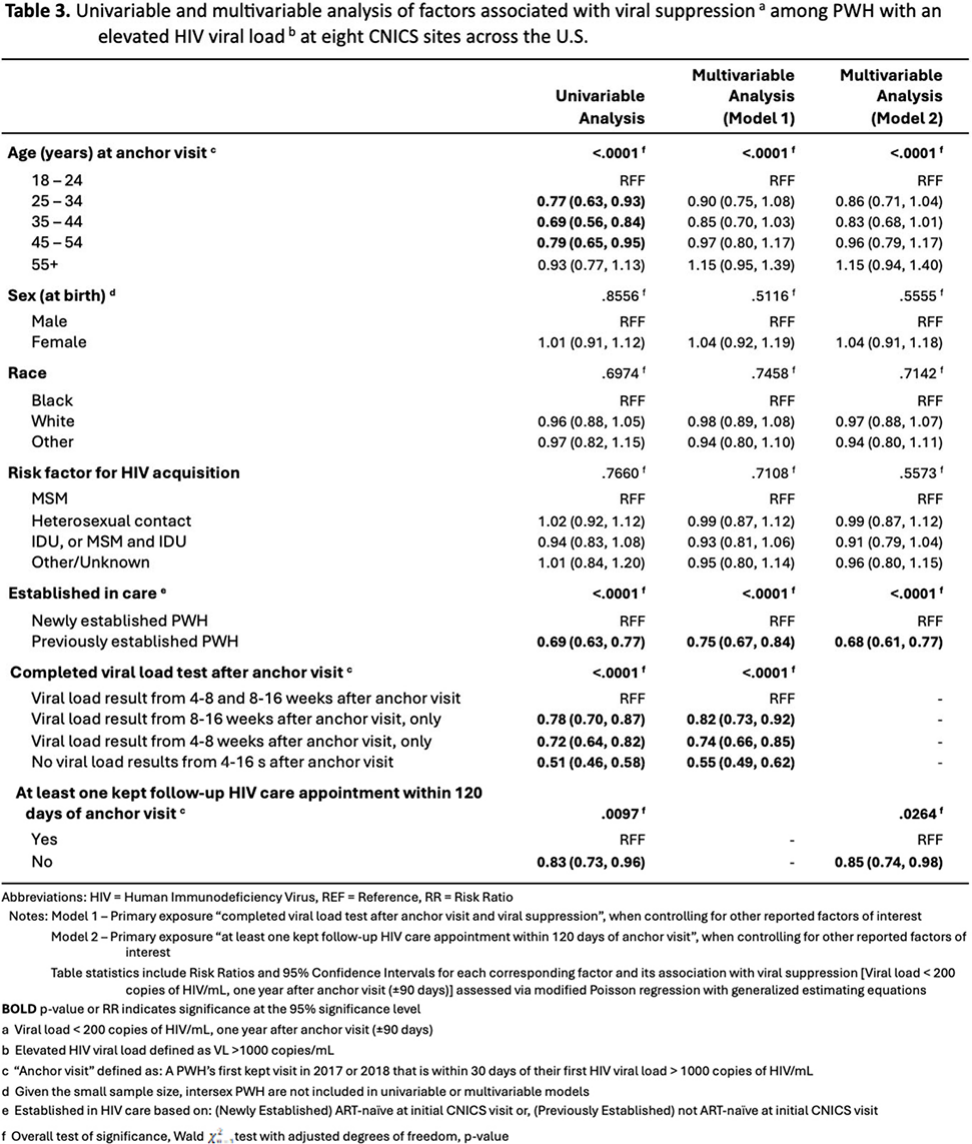

**Results:**

Among 2345 PWH, 11.9% were new to CNICS and 88.1% were already in care. One year from AV, 47% achieved VS. Of the 53% who were unsuppressed, only 39% had confirmatory lab data, with 61% not having any lab data 1 year from AV. Compared to PWH with labs at both 4-8 wk and 8-16 wk, those with no lab visits within 4-16 wk were 0.55 (95% CI 0.49-0.62) times as likely to achieve VS, those with a lab visit 4-8 wk after AV were 0.74 (0.66-0.85) times as likely to achieve VS  and those with a lab visit 8-16 wk after AV were 0.82 (0.73-0.92) times as likely to achieve VS at 1 year. A sensitivity analysis excluding PWH with missing data confirmed these results. PWH with no provider visit within 120 d of AV were 0.85 (0.74-0.98) times as likely to achieve VS at 1 year compared to those with at least 1 visit.

**Conclusion:**

In this large cohort of PWH in routine care across the US, adherence to guideline-recommended early lab monitoring and follow up visits after a VL >1000c/mL was associated with subsequent VS at 1 year. These metrics could serve as early markers of RIC and identify PWH at higher risk of virologic failure than most RIC measures that require 1 year of follow up, allowing for earlier intervention

**Disclosures:**

Katerina Christopoulos, MD, MPH, Janssen: Honoraria Kenneth H. Mayer, MD, Gilead Sciences: Advisor/Consultant|Gilead Sciences: Grant/Research Support|Merck, Inc: Advisor/Consultant|Merck, Inc: Grant/Research Support|Moderna: Grant/Research Support|ViiV Healthcare: Advisor/Consultant|ViiV Healthcare: Grant/Research Support

